# Intraperitoneal administration of NK-92 improves survival in xenografts of early and established ovarian cancer models

**DOI:** 10.1371/journal.pone.0347095

**Published:** 2026-04-20

**Authors:** Paula Marcus, Jenny Warrington, Michael Zhang, Manjunatha Ankathatti-Munegowda, Xing-Hua Wang, Iran Rashedi, Armand Keating

**Affiliations:** 1 Krembil Research Institute, University Health Network, Toronto, Ontario, Canada; 2 Princess Margaret Cancer Centre, University Health Network, Toronto, Ontario, Canada; University of Michigan, EGYPT

## Abstract

Ovarian cancer (OC) is a leading cause of gynecological cancer-related mortality. Management remains challenging as the disease frequently presents with intra-abdominal metastases at diagnosis, standard therapies can be associated with severe complications and disease recurrence is common. While cellular immunotherapy is increasingly investigated as a promising approach, the most effective routes of administration need to be established. We have investigated NK cell therapeutics as a less toxic option and examined the permanent NK cell line, NK-92, as a suitable model. Here, we report two xenograft mouse models with the ovarian adenocarcinoma cell line, SKOV-3, representing early-stage OC and late-stage OC with ascites to comprehensively evaluate the anti-cancer efficacy of different routes of NK-92 administration. Bioluminescence imaging, cell tracking with the IVIS system and animal survival were used to evaluate outcomes. The cells were administered via intraperitoneal (IP), intravenous (IV), or a combination of IP and IV routes. We showed that NK-92 significantly increased animal survival when delivered IP (*p* = 0.009 and *p* = 0.018) or combined IP and IV (*p* = 0.05 and *p* = 0.017) in early and established OC xenografts, respectively, whereas intravenous delivery at similar doses had no effect on survival (*p* = 0.665 and *p* = 0.052) compared with untreated controls. These findings, and our novel models, have potential implications for enhancing the clinical benefit of NK therapy in patients with advanced OC.

## Introduction

Ovarian cancer (OC) has the highest mortality rate of any gynecological malignancy with a 5-year survival of less than 45% and is the fourth leading cause of cancer-related deaths in women worldwide [1]. The disease remains asymptomatic until late stages when intra-abdominal metastases are present, and the cancer is more resistant to therapy. The rate of recurrence is high after standard cytoreductive surgery and adjuvant chemotherapy, and frequently remains confined to the peritoneal cavity [1,2]. Prognosis of refractory disease is poor, and although incorporation of intraperitoneal (IP) chemotherapy shows survival benefit, it can be associated with severe complications [1,3].

While cellular immunotherapy with CAR-T cells has become standard of care for several sub-types of blood cancers, success in treating solid tumors is limited and remains associated with severe side effects, despite increasing research in this area [4]. Natural killer (NK) cells are potentially a more effective alternative due to the ability to target and kill tumor cells without graft-versus-host disease or the serious adverse events observed with T cell therapies. Early studies with autologous NK cells were mostly ineffective [5–8] and clinical benefit was limited with allogeneic peripheral blood derived NK cells [9–11].

The permanent NK cell line, NK-92, established in 1992 from a patient with NK cell non-Hodgkin lymphoma, exhibits higher cytotoxicity against a variety of cancer cell lines compared with primary NK cells and has been extensively investigated in pre-clinical studies and early phase clinical trials [12,13]. We previously conducted a GMP-compliant Phase I clinical trial of dose escalated, repeated intravenous (IV) infusions of irradiated NK-92 to treat patients with refractory blood cancers [14]. We observed only mild adverse events, no cases of cytokine release syndrome or immune-effector cell-associated neurotoxicity syndrome and an overall excellent safety profile, even at very high cell doses (up to 5x10^9^ cells/m^2^ per infusion) or after multiple infusions. We showed preliminary evidence of efficacy, documenting several sustained complete responses despite the use of irradiated NK cells which do not proliferate or persist *in vivo* but retain cytotoxic potency for up to 24 hours [15,16].

In sum, our clinical trial demonstrated the feasibility, safety and efficacy of repeated infusions of irradiated NK-92. Other studies show the highly reproducible cytotoxicity of NK-92 against specific cancer targets and the feasibility of genetically modifying the NK cells [13].

The recent success of CAR-NK cells in treating CD19 + lymphoid malignancies [17] underscores the importance of investigating the NK cell therapeutics of OC. Pre-clinical studies of CAR NK-cells with human OC show enhanced targeting and potency *in vitro* as well as improved survival in *in vivo* models of established OC. Efficacy, however, was evaluated mostly *in vitro*, and in the limited *in vivo* models employing intravenous cell delivery, data on ascites fluid are lacking [18–21].

Early clinical studies of NK cells to treat OC primarily employed intravenous administration [10,22–24]. Clinical trials of chimeric antigen receptor (CAR) NK-cells (from cord blood or induced pluripotent cell sources) for OC are currently ongoing, with two delivering cells via the IP route [ClinicalTrials.gov ID: NCT05922930; NCT06342986]. Overall, data from pre-clinical and clinical studies investigating the different routes of NK cell therapy administration are limited.

Taken together, these studies indicate that NK-92 is a particularly suitable pre-clinical model to investigate NK cellular immunotherapy [12–14].

In this study, we determine optimal anti-cancer efficacy of NK-92 by comparing IV, IP and combined IP and IV routes of administration in two xenograft mouse models of early-stage OC and late-stage disease with ascites, employing IVIS imaging and animal survival.

## Materials and methods

### Cell culture

The human ovarian carcinoma cell line, SKOV-3 stably expressing the firefly luciferase gene was used in all experiments (SKOV-3/Luc; ATCC, Manassas, VA). SKOV-3/Luc were maintained in McCoy 5A media (Invitrogen, Grand Island, NY), supplemented with 10% heat inactivated FBS. NK-92 cells were obtained from Conkwest (Boston, MA, later Nantkwest, San Diego, CA) and cultured in GM1 medium: X-Vivo 10 (Lonza Canada, Mississauga, ON), supplemented with 1.8mM L-serine (Sigma-Aldrich Canada Ltd, Oakville, ON), 0.6mM L-asparagine (Sigma-Aldrich), 3mM L-glutamine (Gibco Canada Inc, Mississauga, ON), 450U/ml IL-2 (Proleukin, Chiron, QC, Canada), and 2.5% human serum (Lonza). Cells were maintained at 37°C in a humidified incubator supplied with 5% CO_2_.

### Chromium release assay

NK-92 cytotoxicity against SKOV-3 and K562 target cells was tested using the chromium release assay (CRA), as previously described [18]. Target cells were diluted to 1x10^6^ in 200 μl AIM-V media and labelled with 100 μCi of Na_2_^51^CrO_4_ for 2 h. Cells were washed twice and resuspended at a final concentration of 10,000 cells/100 μl in AIM-V serum free media (Invitrogen). Target cells were plated at 10,000 cells per well into a 96 well U-bottom plate, and effector cells were added at final a effector:target ratio (E:T) ranging from 0.5:1 to 20:1. To determine the mechanism of cytotoxicity, EGTA (1mM, Sigma Aldrich) was added to the wells in some assays. Plates were centrifuged for 5 minutes at 500 rpm and incubated at 37°C for 4, 18 or 24 h. Supernatant was removed, placed in a collection tube and the radioactivity was quantified using a gamma-counter (Wallac Wizard 3 1480 Automatic Gamma Counter, PerkinElmer Livesciences, Woodbridge, ON).

### Ovarian cancer xenograft model

Two OC xenograft models were generated by injection of 2x10^6^ SKOV-3/Luc cells IP in 6–8-week-old NOD/SCID/γ_c_^null^ (NSG) mice on day zero. In the early tumor model, mice were treated with 6 doses of NK-92 on days 3, 5, 7, 10, 12 and 14. In the established disease tumor model employed to investigate the effect of ascites fluid on NK-92 treatment, 30 days (day 30) after the injection of SKOV-3/Luc at day zero mice were grouped so that the average bioluminescence (tumor burden/spread) was evenly distributed among groups. In this model, NK-92 injections were given over a 16-day period starting at day 38 post tumor initiation. NK-92 cells were irradiated prior to each injection (1000 cGy) and administered either IV (15x10^6^), IP (15x10^6^) or a combination of IV + IP (7.5x10^6^ per route) in 200 ul saline. Before injection, the cytotoxicity of NK-92 was tested by CRA to ensure optimal activity. Control animals were left untreated. Tumor progression was monitored by weekly bioluminescence imaging and mice were sacrificed when humane endpoints were reached.

The effect of single or multiple NK-92 doses was determined in the early SKOV-3 xenograft model. Briefly, mice were given SKOV-3/Luc followed by either 1 dose (day 3) or 5 doses (days 3, 5, 7, 10 and 12) of NK-92 (15x10^6^). Mice were monitored regularly and sacrificed at a humane endpoint. All procedures were approved by the institutional Animal Care and Use Committee of the University Health Network.

Animals were euthanized when humane endpoints were reached, which included abnormal posture, failure to groom, lethargy, impaired ambulation or paralysis, weight loss exceeding 20% normal body weight, abnormal vocalization, persistent anorexia, haemorrhagic discharge, abdominal distention causing distress/pain, dyspnea, cardiovascular collapse, persistent self trauma, tumour size exceeding 1.5 cm, ulcerated tumours, pre-moribund, and moribund (severe depression, unable to respond to external stimuli, non-ambulatory, inability to right itself). Once animals reached endpoint criteria, they were euthanized immediately. Euthanasia was performed by inducing the mice with isoflurane as a general anaesthetic, followed by CO_2_ overdose. The experiment duration was 130 days. For the two xenograft models involving survival and cell tracking, 100 mice were used. An additional 24 mice were used to test single versus multiple NK-92 doses. These mice were obtained from the Cancer Stem Cell Colony at the University Health Network. Animals were monitored twice daily for health and behaviour, and no animals died before meeting euthanasia criteria. All research staff received training in animal anaesthesia, animal surgery, experimental procedures, and monitoring/nursing. All animal welfare considerations were taken to minimize suffering and distress, including frequent monitoring and immediate euthanasia at endpoint. All animal procedures were performed in compliance with the institute Animal Care Committee and performed in accordance with the Canadian Counsel on Animal Care Guidelines.

### Bioluminescence imaging

Before injection, luciferase activity was evaluated in SKOV-3 cells. Cells were plated at 1000–3000 cells/well in a 24-well plate and left to adhere overnight. D-luciferin (Perkin Elmer) was diluted in complete medium to a final concentration of 150 μg/ml, and 200 ul was added to each well. Images were taken every 30 seconds for 5 minutes, using the IVIS Spectrum system (Xenogen, Alameda CA).

For *in vivo* bioluminescence analyses, mice were injected IP with 150 μg/g D-luciferin in PBS and anesthetized by isoflurane inhalation. Images were collected 15 minutes after injection using the IVIS system with acquisition times ranging from 0.5 second to 1 minute. Bioluminescence images were analysed using Living Image software (Xenogen), and data were expressed as photon flux (photons/s/cm^2^/steradian) or total area (cm^2^) measured over the region of interest (ROI).

### Cell tracking

NK-92 cells were tracked after injection in both early and established OC models. SKOV-3/Luc cells were administered as described earlier. NK-92 cells were washed and labelled in 3.5 μg/ml DiR buffer for 30 minutes as per the manufacturer’s guidelines (Perkin Elmer). After staining, a single dose of NK cells was given either 3 or 38 days (in the early and established models, respectively) after tumor initiation via IP, IV or IP + IV injection (total of 15x10^6^ cells). Mice were imaged at 4, 24, 48 and 72 hours post NK treatment using the IVIS system. First, bioluminescence imaging was performed as previously described in the supine and prone positions and then, fluorescent images were collected using excitation at 710 nm and emission at 760 nm. After 72 hours, organs were harvested from two mice per group and imaged for DiR labelling. Total flux (photons/s) was calculated for each organ.

### Statistics

Statistical analysis was conducted using Prism 5 software (Graphpad, San Diego, CA). *In vivo* data were analysed using two-way Anova with multiple comparisons between column means to determine statistical differences between treatment groups. Survival probabilities were analysed using the Weibull test. Data are presented as mean ± SEM. A p-value of <0.05 was considered statistically significant.

## Results

### NK-92 is cytotoxic against SKOV-3

We evaluated the cytotoxic effect of NK-92 on SKOV-3 using the chromium release assay (CRA). In a standard 4h assay, NK-92 cells showed a limited cytolytic ability against SKOV-3 at all E:T ratios compared with the control leukemia cell line, K562 (e.g., 9% in SKOV-3 vs 65% in K562 at E:T 10:1, Fig 1A). NK-92 cytotoxicity against SKOV-3, however, increased with longer incubation times (e.g., 25% at 18h and 45% at 24h at E:T 10:1, Fig 1B). The longer incubation time did not significantly change K562 lysis by NK-92 (e.g., 76% at 18h and 77% at 24h at E:T 10:1). We further tested NK-92 cytotoxicity against these two cell lines in the presence of the calcium chelating agent, EGTA. Addition of EGTA significantly reduced NK-92 cytotoxicity against K562 (12.6 fold reduction at 18h and 7.3 fold reduction at 24h, E:T 5:1) but only partially decreased NK-92 cytotoxicity against SKOV-3 (3.1 fold at 18h and 3.8 fold at 24h, E:T 5:1, Fig 1C). This suggests that different pathways are involved in NK cell-mediated killing of these two cancer lines. Calcium-dependent toxic granule exocytosis is likely the primary pathway involved in the killing of K562, whereas alternative death pathways are more likely activated against SKOV-3 and result in the formation of apoptotic bodies. These data indicate that NK-92 targets and kills SKOV-3 in a dose- and time-dependent manner *in vitro*.

**Fig 1:**
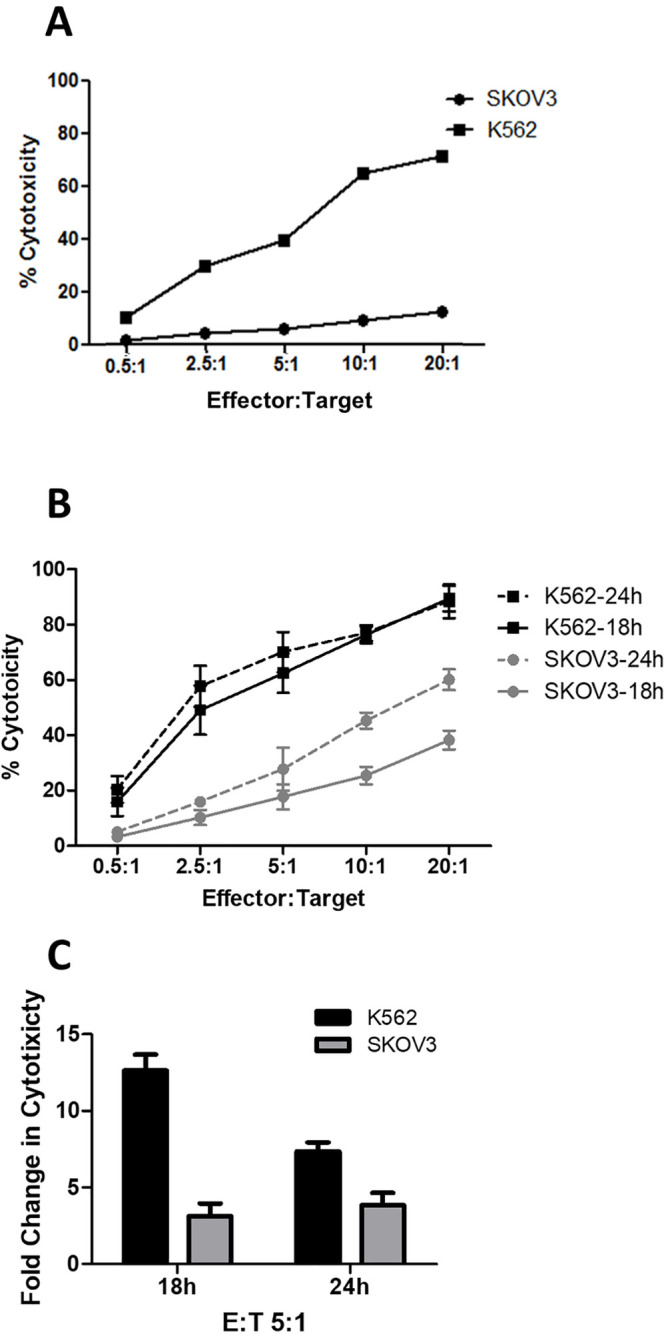
NK-92 is cytotoxic against SKOV-3. (A) In a 4h chromium release assay, NK-92 lysed SKOV-3 in a dose-dependent manner; the cytotoxicity was lower than the leukemic target cell line, K562. (B) NK-92-mediated killing of SKOV-3 was increased after 18h and 24h incubation. (C) In the presence of EGTA, NK-92 cytotoxicity was less affected against SKOV-3 than K562 cells. Data are presented as mean ± SEM, n = 3 independent experiments.

### IP or IP + IV delivered NK-92 increased survival in a model of early OC

**Fig 2:**
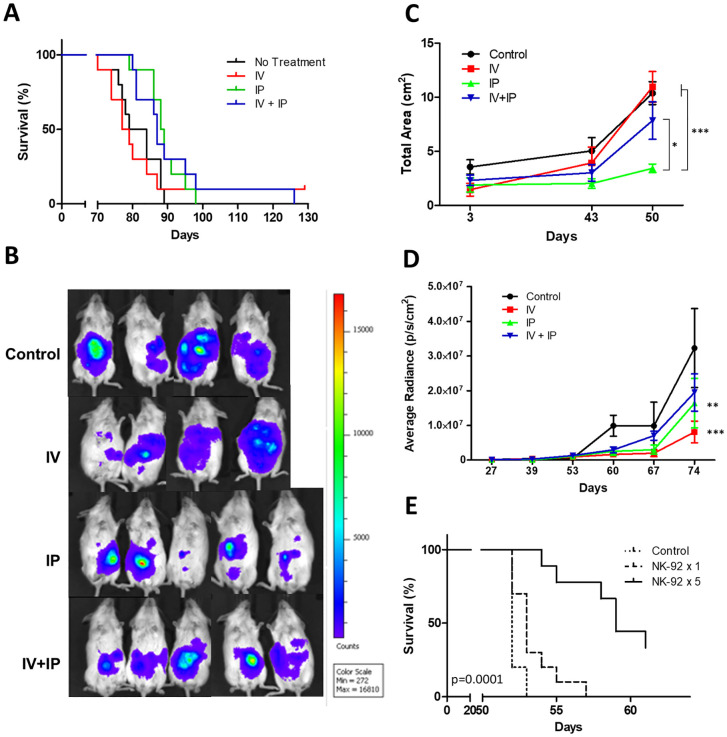
IP administration of NK-92 enhanced survival and reduced ascites formation in a SKOV-3 xenograft model of early ovarian cancer. **(A)** There was a significant increase in survival after treatment via IP (p = 0.009) or IV + IP (p = 0.050) injections compared with untreated controls, n = 9-10. **(B)** *In vivo* bioluminescence imaging of SKOV-3/Luc mice in each treatment group. **(C)** IP administration of NK-92 resulted in a significant decrease in the distribution area of SKOV-3/Luc signal compared with controls or mice that received NK-92 via IV or IV + IP injections, n = 5. **(D)** Treatment with IV or IP administered NK-92 resulted in a decrease in the average radiance compared with control mice, n = 5. **(E)** Multiple IP injections, but not a single IP dose of NK-92 improved survival, n = 8. Data are presented as mean ± SEM (*p < 0.05, **p < 0.01, ***p < 0.001).

To determine the optimal route of NK-92 administration for the treatment of OC, we compared the efficacy of IV, IP or IV + IP delivery of NK-92 in a SKOV-3/Luc xenograft model of ovarian cancer. NK-92 delivered either IP or IV + IP significantly increased both median and overall survival compared with untreated controls (median 88.5 and 87 days in IP and IP + IV groups vs 81.5 days in controls; p = 0.0093 and p = 0.050, respectively). There was no effect on survival after IV delivery of NK cells compared with the control group (median 78 days; p = 0.665; Fig 2A). Using whole body IVIS imaging, we examined the area of SKOV-3/Luc distribution as an indicator of ascites fluid accumulation in the peritoneal cavity, in which tumor cells can spread and metastasize. We found a significant decrease in the area of bioluminescence signal in mice treated IP compared with other groups after 50 days. The difference was more significant in comparison with untreated controls (p < 0.001) and IV treated mice (p < 0.001) than mice treated via IP + IV (p < 0.05, Figs 2B and 2C). This suggests that direct administration of NKs into the peritoneal cavity reduced ascites formation in these mice, likely as a result of higher local NK cell concentration in the peritoneal cavity. The average bioluminescence signal was significantly decreased after either IV (p < 0.001) or IP (p < 0.01) treatment with NK-92 (Figs 2B and 2D). The latter indicates that IP or IV administration of NK cells may be similarly effective in reducing cancer cell burden. Nevertheless, the presence of solid tumors (with maximal bioluminescence intensity, Fig 2B) within the peritoneal cavity of mice in all groups indicates that the NK-92 dose and route of administration employed were unable to result in disease-free status.

### Multiple but not single IP doses of NK-92 improve survival

Following the observed survival improvement after IP treatment, we tested the effects of single versus multiple (x5) IP injections of NK-92 on survival. We found no statistically significant survival benefit after a single dose of NK-92 (p = 0.0541) but, as shown earlier, multiple doses of IP-delivered NK-92 increased survival compared with untreated controls and single-dose treated mice (p = 0.0001, Fig 2E).

### IP or IP + IV delivery of NK-92 significantly increases survival in mice with established OC

**Fig 3:**
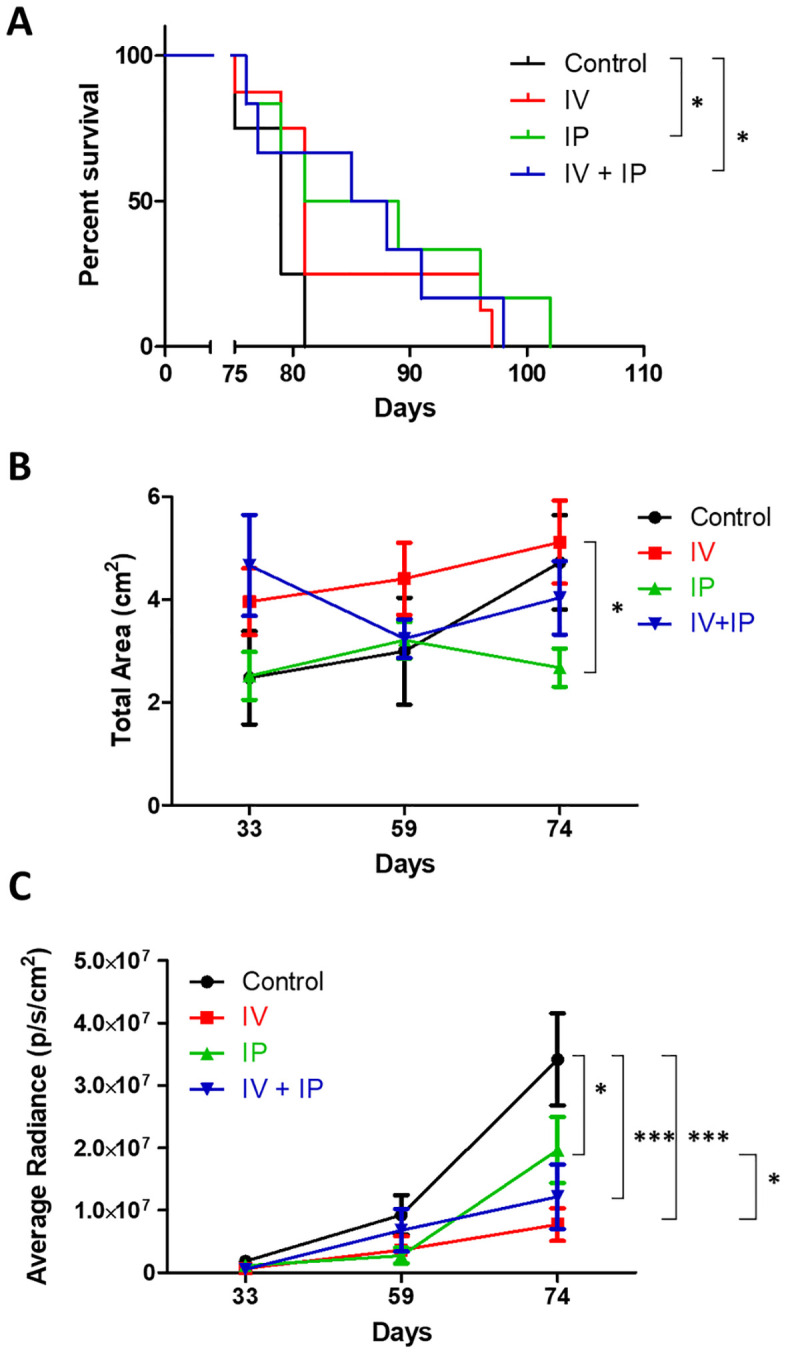
IP or IV+IP administration of NK-92 enhanced survival in a model of established ovarian cancer. **(A)** Survival increased significantly after treatment via IP [*χ² (1, N = 4,6) = 5.57, p = 0.018, 95% CI (−0.13, −0.03)]* or IV + IP [*χ² (1, N = 4,6) = 5.69, p = 0.017, 95% CI (−0.11, −0.03)*] injections compared with untreated controls. **(B)** IP administration of NK-92 resulted in a significant decrease in the area of SKOV-3/Luc signal compared with the IV-injected group. **(C)** Treatment with NK-92 resulted in a decrease in the average radiance in all groups compared to control animals 6 weeks post-treatment, n = 4-8 *p < 0.05, **p < 0.01, ***p < 0.001).

We next investigated the efficacy of IP injection in our novel model of established disease and associated ascites. In this model, NK-92 was injected 5 weeks after tumor initiation, when ascites fluid could be first detected in the peritoneal cavity. The median survival of untreated control mice increased after treatment with IP and IV + IP NK-92 (from 79 days to 85 and 86.5 days, respectively) whereas the median survival in IV treated mice remained closer to the control group (81 days). Using the Weibull distribution survival analysis we found a significant increase in survival after IP and IV + IP treatments (p = 0.018 and p = 0.017, respectively) whereas IV treatment showed no significant survival benefit (p = 0.052) compared with the control group (Fig 3A).

Similar to the early OC model, bioluminescence analyses indicated a decrease in the total area of SKOV-3/Luc signal in the IP treated group, suggesting a lower quantity of ascites fluid in the peritoneal cavity in this cohort. The difference was not statistically significant compared with untreated controls but was significantly lower than the IV treated group 6 weeks post-treatment (p < 0.05, Figs 3B and 3C). This could be attributed to the higher ascites signal present in the established disease model prior to therapy, likely making treatment with IV NK-92 less effective. There was a significant decrease in average bioluminescence after treatment with NK-92 (Fig 3D), indicating a decrease in tumor burden in all groups after treatment, which was more significant in IV and IV + IP treated groups (p < 0.001).

### NK-92 primarily localizes to liver and spleen and co-localize with solid tumors

**Fig 4:**
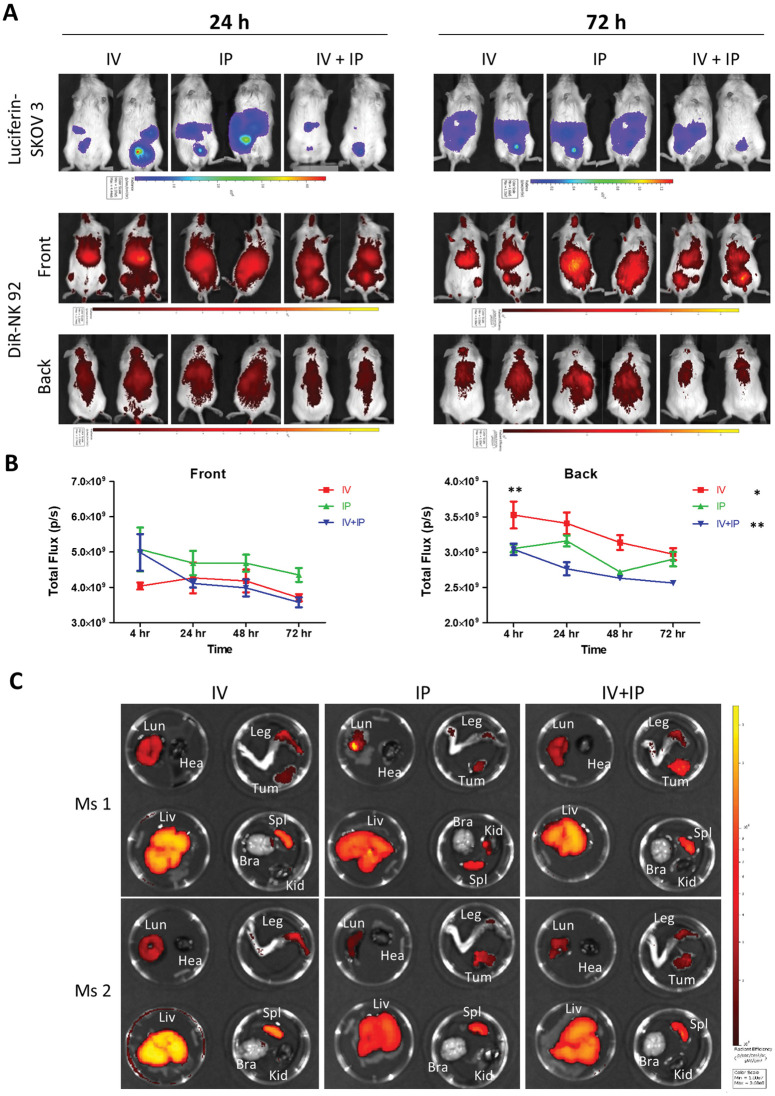
NK-92 mainly localizes to liver and spleen and co-localize with tumor cells. NK cells localized mostly to the peritoneal cavity after IP administration in a model of established OC. **(A)** Bioluminescence and DiR signals, respectively, indicate SKOV-3 and NK-92 *in vivo*. IP delivered NK-92 remained primarily within the peritoneal cavity whereas IV-injected cells accumulated around the liver at 24 h and both liver and thoracic region after 72 **h. (B)** Quantification of DiR signal indicated a significant increase in DiR signal shortly after IV injection in the prone position (** p < 0.01). There was a significant decrease in DiR signal in the IV and IV + IP groups over time (*p < 0.05, ** p < 0.01 comparison between 4h and 72 **h)**, n = 4. **(C)** Analysis of DiR signal in harvested organs 72 h after injection showed NK-92 predominantly located in the liver and spleen and was also detectable in solid tumors. Bra, Brain; Hea, Heart; Kid, Kidney; Liv, Liver; Lun, Lung; Spl, Spleen; Tum, Tumor.

To better understand the *in vivo* distribution of NK-92 cells post-injection that might have contributed to the increased survival of tumor-bearing animals after IP infusion, we labelled NK-92 with the fluorescent membrane dye, DiR, and tracked the injected cells for 72 h. In mice injected IP with DiR-stained NK-92, cells were more confined to the abdomen whereas after IV injection, the signal was more scattered across the body. NK cell dispersion in the IV + IP delivered group exhibited a pattern consistent with a combination of the IV and IP cohorts (Fig 4A and S2 Fig). Comparison of signals from the ventral plane showed no significant difference in DiR signal measured at any of the time points and no decrease in the total signal over the same period (4–72 h), suggesting no significant change in NK-92 cell numbers (Fig 4B and S2 Fig). Signal viewer from the dorsal position showed significantly higher DiR NK-92 signal 4 h after IV injection in both early and established OC models (p < 0.05 compared with IP in the early OC model, S2 Fig, and <0.01 compared with IP and IV + IP in the established OC model, Fig 4B), most likely due to migration of the cells to the liver and lungs. In the established OC model, DiR signal was similar 4 h and 72 h post-injection in the prone position in IP group (suggesting no significant redistribution of cells) whereas both IV and IV + IP groups had a significant decrease in signal over time in this position (p = 0.050 and p = 0.0027, respectively, Fig 4B).

At 72 h, organs were collected from two mice per treatment group for a closer examination of DiR NK-92 cells (Fig 4C and S2 Fig). In all animals, including the untreated controls in both early and established OC models, the highest DiR signal was in the liver, with high numbers of cells also present in the spleen and legs. IP injection of DiR-stained NK-92 resulted in a distinct increase in signal in the lungs in the established OC model, not observed in the early OC model or in tumor-free controls (S3 and S4 Figs). This could be due to increased ascites fluid and breakdown of barriers in the established cancer facilitating systemic migration of NK-92. In the established OC model, we were able to remove detectable solid tumors and assess DiR-stained NK-92 accumulation. Of the two mice studied per group, only one in the IV group had a solid tumor present. DiR signal was detected in all solid tumors collected, with the highest intensity observed after IV + IP delivery (S4 Fig), suggesting increased NK-92 co-localisation with tumors in this group. With the exception of the lung in the IP group, there were no differences in the DiR signal pattern between tumor-bearing mice and tumor-free cohorts injected with DiR-stained NK-92 (S4 Fig). Overall, these data indicate a trend for reduced numbers of NK-92 cells in the organs after IP delivery and suggest that the observed improvement in survival after IP injection of NK-92 may result from increased NK cell numbers within the peritoneal cavity, thereby enhancing E:T ratio and tumor cell killing.

## Discussion

In this study, we evaluated different routes of administration of NK-92 in human ovarian cancer xenograft mouse models to assess effects on tumor burden and survival. We found that multiple doses (5x) of cells delivered via IP or combined IP and IV injections resulted in a significant reduction of tumor burden and increased experimental animal survival (p = 0.018 and p = 0.017, respectively). No significant tumor reduction was observed with IV injections, or single dose treatments via any delivery method. Complete tumor regression was not achieved with any treatment. Nevertheless, the mean duration of survival was increased in mice treated with 5 doses of NK-92 (15x10^6^) delivered IP or a combination of IP and IV injection.

Despite improvements in the treatment of primary ovarian cancer, effective management of relapsed/refractory disease is lagging. Nonetheless, major advances in the management of relapsed lymphoid malignancies with chimeric antigen receptor (CAR)-T and CAR-NK cell therapy [17,25] has stimulated further interest in conducting similar studies to treat solid tumors [26]. Previous trials of NK cells to treat OC have been restricted to IV administration [10,22–24]. More recent studies with CAR-NK cells have employed the IP route but data are limited (ClinicalTrials.gov ID: NCT05922930). Moreover, evaluation of the potential importance of routes of cell administration on treatment outcomes is infrequently addressed in studies with pre-clinical xenograft models [18,21,27]. While previous studies have shown that IP delivery of NK cells enhances target cell cytotoxicity [28], our study is the first to compare the three methods of NK cell delivery via IP, IV and combined IP and IV routes and to determine the effect on ascites accumulation, tumor burden and survival of animals.

We used two xenograft models of SKOV-3, a human OC line to generate an early-stage and an established intraperitoneal late-stage OC model. The SKOV-3 line is able to generate ascites in the NSG mouse [29]. These mice were subsequently treated with successive doses of NK-92 via different delivery methods to assess efficacy against tumor burden and potential ascites fluid accumulation. SKOV-3 was transduced to stably express firefly luciferase to enable *in vivo* tracking. Using IVIS imaging, intraperitoneal distribution of the luciferase signal indicated formation of ascites fluid. SKOV-3 also exhibits partial resistance to NK-mediated killing (as reflected in our 4h CRA data) to enable the detection of potentially small differences due to the different administration routes tested. We observed a time-dependent increase in NK-92 cytotoxicity against SKOV-3 indicating the effective targeting of SKOV-3 *in vivo*. The time-dependent increase in cytotoxicity against SKOV-3 we observed requires further elucidation but may involve mechanisms other than granule exocytosis, including the Fas/Fas Ligand and TRAIL death receptor signaling pathways, as determined or proposed by others [30].

We demonstrated that IP, or a combination of IV + IP administration of NK cells is superior at targeting OC compared with IV infusion. We found that both IP and IV + IP administration of NK-92 significantly increased survival of both early and advanced stage OC xenograft models. The superior results of cohorts receiving NK cells that included the IP route are consistent with an expected higher local effector to target cell ratio in the peritoneal milieu. We also found reduced abdominal ascites after IP treatment as determined by the area of bioluminescence signal. While this observation was not reflected in differences in tumor burden among the groups, it is a relevant metric, as ascites accumulation is associated with increased intraperitoneal cancer dissemination and is an important factor in the management of OC [31–33].

A critical consideration in cellular therapy for solid tumors is efficacy of trafficking to the tumor site. Our data are consistent with other studies showing that modified NK cells delivered IV, accumulate within the spleen and liver [28,34], whereas IP-injected NK cells are found within the peritoneal cavity in high numbers [28,34,35]. A drawback of previous experimental models is the injection of NK cells within 7 days of cancer initiation. Given that NK migration relies, in part, on secreted cancer cell signals, it is possible that a different rate of effector cell trafficking occurs in an already established cancer in which normal signalling is disrupted [36]. We found that one animal treated with IP infusion had increased NK cells which had migrated to the lung compared with the control, possibly due to increased access to the systemic circulation associated with accumulation of ascites fluid. We also established that NK-92 trafficked to the liver within the first 4 h after administration and remained there for at least the next 72 h.

The choice of delivery method should also be based on practical considerations. In our study, treatment with IP-delivered NK cells resulted in a decrease in accumulation of ascites whereas IV treatment decreased tumor mass. Compared with intraperitoneal access, IV delivery is likely to enhance NK penetration of vascularized tumors leading to more effective cancer cell targeting. This is supported by our data showing reduced tumor burden (BL signal intensity) in IV-treated mice in both OC models of early and established disease (Figs 2D and 3D, respectively). The increased DiR signal in harvested tumors also suggests increased NK-92 co-localization within solid tumors in mice with established OC after IP + IV treatment. The latter may account for the observed improvement in survival in this group, which was more pronounced than in mice with early OC. It is also conceivable that for some metastatic tumor patterns, a combination of both IV and IP routes may be more effective.

IP injection may promote tumor clearance by increasing local E:T ratios, thereby obviating the need for repeated doses of NK cells. Hermanson *et al.* found that a single IP injection of induced pluripotent stem cell-derived NK cells was sufficient to significantly increase survival in an OC model [37]. In our study, we did observe a small, although not significant, increase in survival with a single dose of IP-administered NK-92. However, it is noteworthy that we found a significant increase in survival after multiple doses of IV + IP infusion of NK-92 cells (half a full dose per route) in both the early and established OC models. Given that IV administration alone conferred no survival benefit in our studies, the increased survival of the IV + IP group could be attributable to the IP portion of the treatment which contained cell numbers lower than the doses commonly reported for the IV infusion of NK cells. In the early OC model, the difference between NK cell numbers delivered intraperitoneally in the IP and IP + IV groups was reflected in better survival outcome in the former group compared with untreated controls (Fig 2). A similar dose effect was observed in a clinical trial by Yang *et al.* in which patients who received repeated injections of higher doses of *ex vivo* expanded NK cells tended to have better outcomes than those treated with a single injection of lower dose [23]. Our data suggest that a combination of cell dosage and delivery route affects treatment outcome and better results may be obtained by optimizing these protocols.

It is noteworthy that several recent clinical studies show the feasibility of IP administration of immunotherapy, including repeated dosage, in patients with advanced ovarian cancer and ascites using indwelling peritoneal catheters, providing further validation for our mouse model [38–41].

In conclusion, the *in vivo* findings with our xenogeneic models show a significant overall survival benefit of IP administration of NK-92 compared with IV injection. Given however, that we did not demonstrate complete tumor regression in our study, further dose-finding experiments are required to optimize the treatment protocol and achieve better outcomes, including the documentation of disease-free survival. We anticipate additional improvements using our xenograft murine models to evaluate CAR-NK cells that in other pre-clinical studies have shown enhanced cytotoxicity and fewer off-target effects [18–20,42,43].

## Supporting information

S1 Fig Schematic representation of the experimental design. In the early ovarian cancer model (top), NK-92 cells were injected in 6 doses starting at 3 days after intraperitoneal (i.p.) injection of SKOV-3/Luc cancer cells in NSC mice. In the established ovarian cancer model (bottom), treatment with NK-92 cells was initiated 5 weeks after tumor initiation. Tumor progression was monitored weekly by bioluminescence imaging.
(TIF)

S2 Fig NK cells primarily localize to the peritoneal cavity after IP administration. (A) Bioluminescence and DiR signals indicating SKOV-3 cancer cells and NK-92, respectively, 24 h and 72 h after administration. IP delivered NK-92 cells are primarily located within the peritoneal cavity of animals while IV delivered cells are more dispersed across the body. (B) Quantification of DiR signal in the supine and prone positions. (C) DiR NK-92 cells were detectable in organs collected 72 h after injection, n = 2. Cells were primarily located in the liver and spleen in all groups. IP, intraperitoneal; IV, intravenous; Hea, Heart; Kid, Kidney; Liv, Liver; Leg, Leg; Lun, Lung; Spl, Spleen.
(TIF)

S3 Fig Quantification of DiR NK-92 in organs 72 h after administration in the early ovarian cancer model. Total DiR signal from each organ was quantified in two animals per group. In all treatment groups, the highest NK-92 frequency was observed in the liver, followed by the spleen and lungs. IP delivery resulted in lower numbers of cells in the lungs compared to IV and IP + IV groups. There was a trend for decreased accumulation of NK-92 cells in organs after IP injection compared to IV and IP + IV groups. IP, intraperitoneal; IV, intravenous.
(TIF)

S4 Fig Quantification of DiR signal in organs 72 h after DiR NK-92 administration in the established tumor model. Total DiR signal was quantified in two animals per group. The results from 1 tumor-free control (ctrl) animal is shown per group. The highest NK-92 presence was detected in the liver. There was a trend towards an increased presence of NK-92 cells in the spleen after IV injection compared with control. DiR NK-92 signal was found in the tumors of all three treatment groups.
(TIF)

S1 File Supporting information file. Minimal data set containing underlying data.
(XLSX)
