# Supplementary figures and images for: Intraperitoneal administration of NK-92 improves survival in xenografts of early and established ovarian cancer models
Source: PLoS One. 2026 Apr 20;21(4):e0347095. doi: 10.1371/journal.pone.0347095 (PMC13095011; doi:10.1371/journal.pone.0347095)

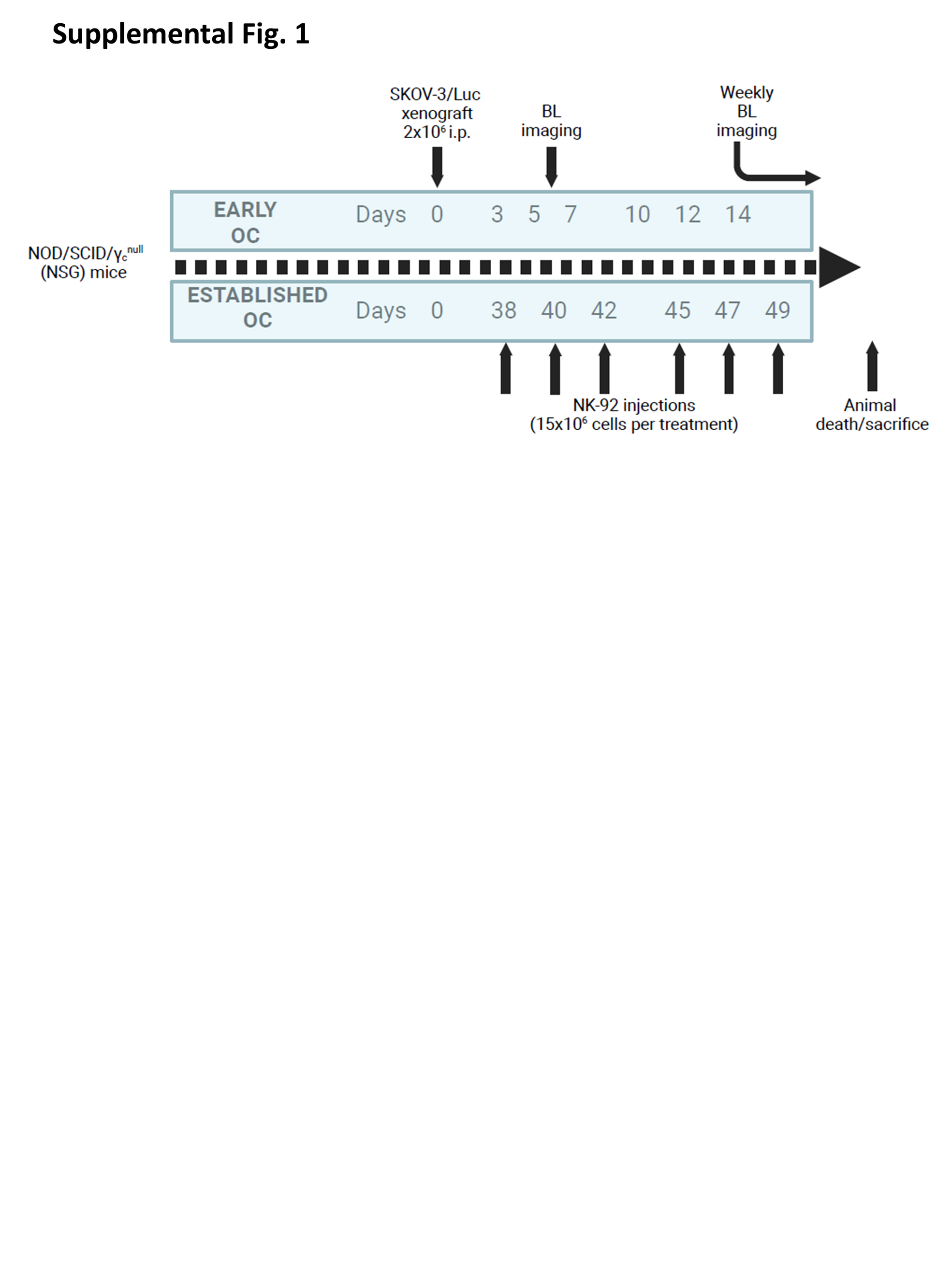

Supplement: S1 Fig — In the early ovarian cancer model (top), NK-92 cells were injected in 6 doses starting at 3 days after intraperitoneal (i.p.) injection of SKOV-3/Luc cancer cells in NSC mice. In the established ovarian cancer model (bottom), treatment with NK-92 cells was initiated 5 weeks after tumor initiation. Tumor progression was monitored weekly by bioluminescence imaging. (TIF) [file pone.0347095.s001.tif]

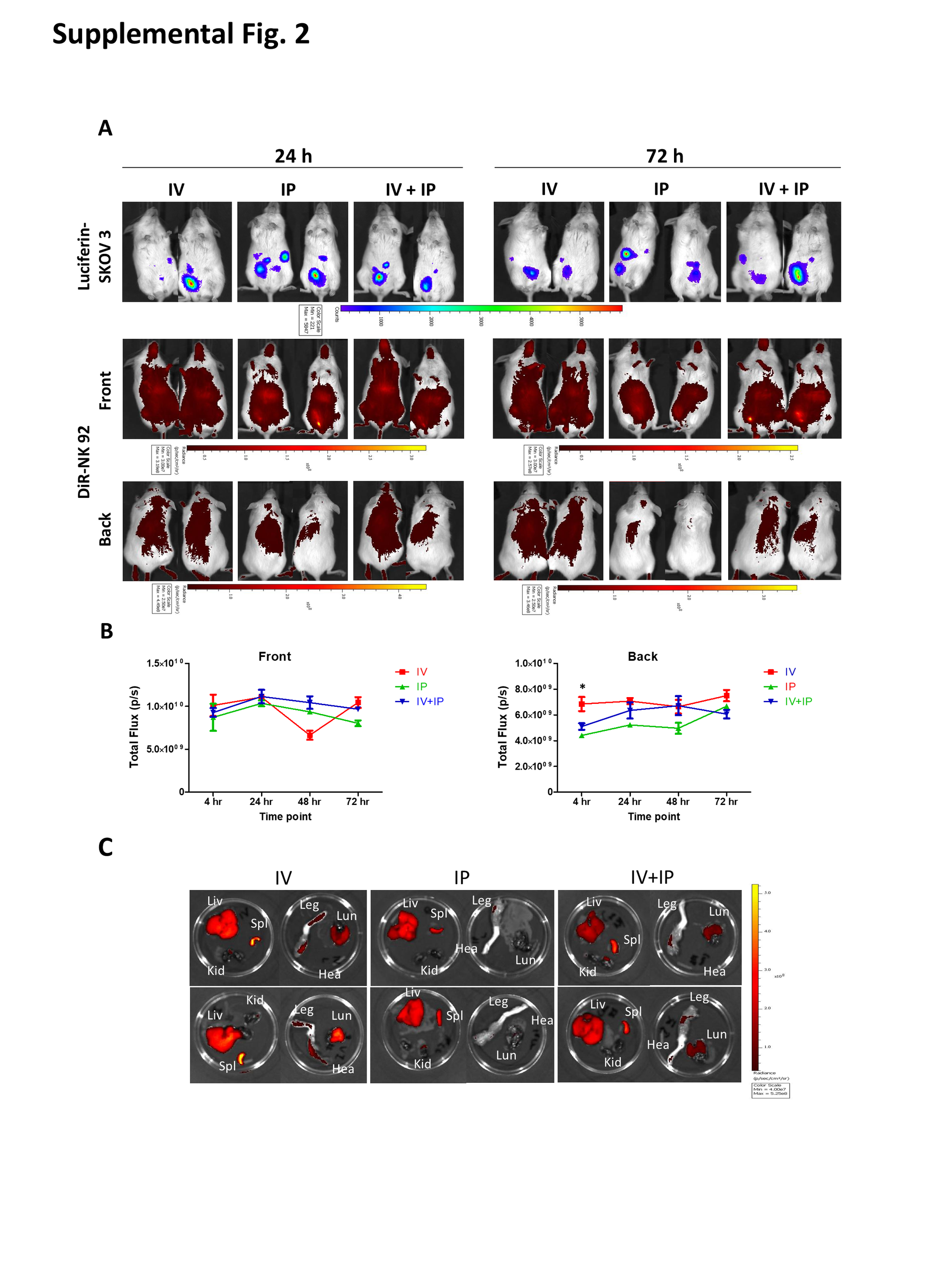

Supplement: S2 Fig — (A) Bioluminescence and DiR signals indicating SKOV-3 cancer cells and NK-92, respectively, 24 h and 72 h after administration. IP delivered NK-92 cells are primarily located within the peritoneal cavity of animals while IV delivered cells are more dispersed across the body. (B) Quantification of DiR signal in the supine and prone positions. (C) DiR NK-92 cells were detectable in organs collected 72 h after injection, n = 2. Cells were primarily located in the liver and spleen in all groups. IP, intraperitoneal; IV, intravenous; Hea, Heart; Kid, Kidney; Liv, Liver; Leg, Leg; Lun, Lung; Spl, Spleen. (TIF) [file pone.0347095.s002.tif]

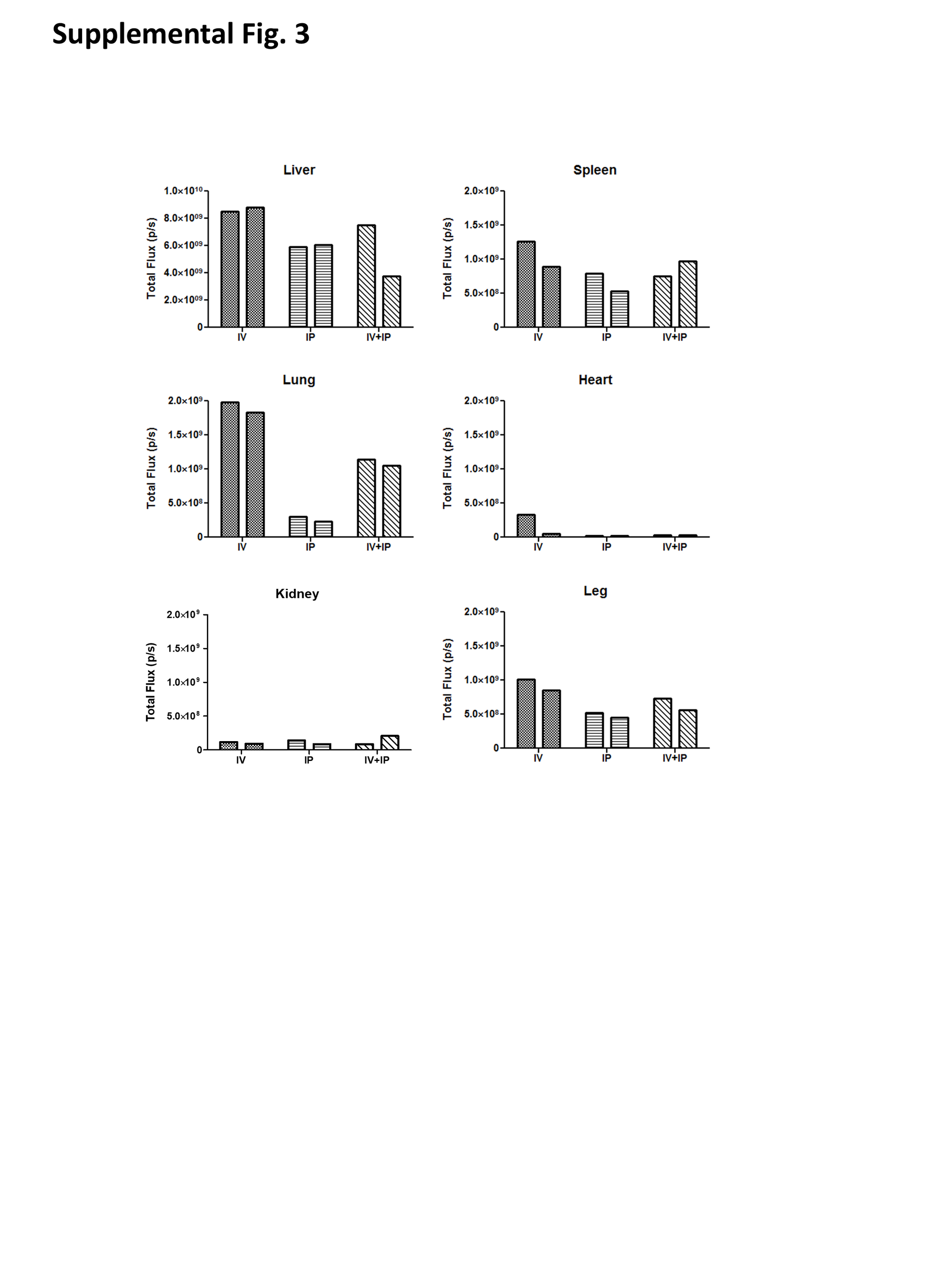

Supplement: S3 Fig — Total DiR signal from each organ was quantified in two animals per group. In all treatment groups, the highest NK-92 frequency was observed in the liver, followed by the spleen and lungs. IP delivery resulted in lower numbers of cells in the lungs compared to IV and IP + IV groups. There was a trend for decreased accumulation of NK-92 cells in organs after IP injection compared to IV and IP + IV groups. IP, intraperitoneal; IV, intravenous. (TIF) [file pone.0347095.s003.tif]

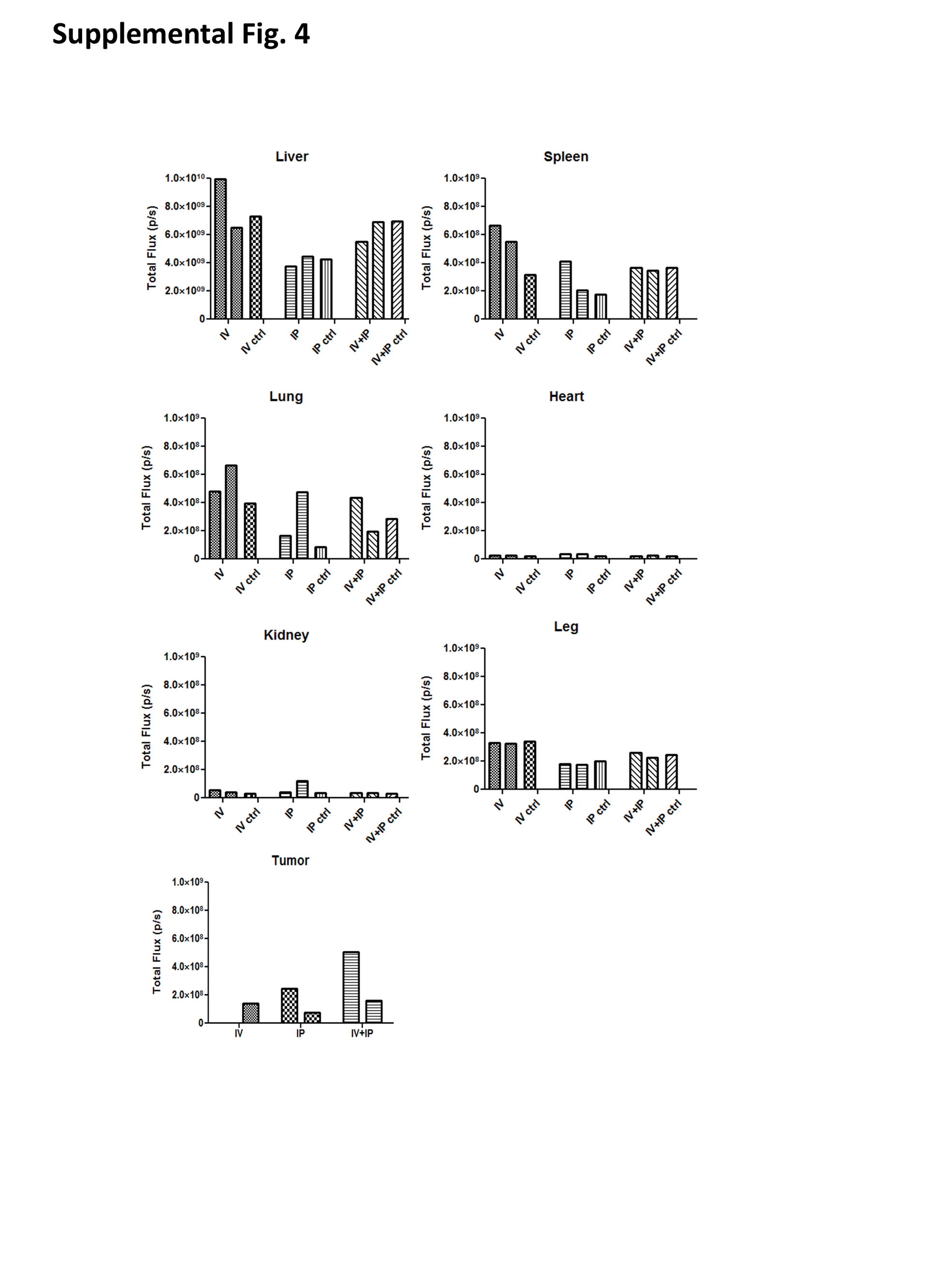

Supplement: S4 Fig — Total DiR signal was quantified in two animals per group. The results from 1 tumor-free control (ctrl) animal is shown per group. The highest NK-92 presence was detected in the liver. There was a trend towards an increased presence of NK-92 cells in the spleen after IV injection compared with control. DiR NK-92 signal was found in the tumors of all three treatment groups. (TIF) [file pone.0347095.s004.tif]
